# Heritable and experience-dependent cortical traits of reading ability

**DOI:** 10.1093/cercor/bhag125

**Published:** 2026-08-22

**Authors:** Mikael Roll

**Affiliations:** Centre for Languages and Literature, Lund University, Helgonabacken 12, 223 62 Lund, Sweden

**Keywords:** anterior temporal lobe, auditory cortex, cortical microstructure, heritability, reading

## Abstract

Reading ability emerges from interacting phonological and semantic skills. However, the cortical microstructure supporting these traits remains unclear. Multimodal cortical parcellation and diffusion analysis were performed in 1,068 participants, including a genetically informed twin subsample. We identified auditory and anterior temporal subregions predicting oral reading scores. In the left auditory cortex, myelin in a paralemniscal (belt) area was heritably linked to reading, whereas thickness in the lemniscal (core) region reflected experience-dependent contributions. Bilateral anterior temporal lobe (ATL) surface areas were genetically associated with reading and predicted semantic performance, providing novel cortical evidence for heritable semantic substrates of literacy. The left inferior fronto-occipital fasciculus and a left ATL region connected with the arcuate fasciculus predicted reading scores independent of genetic influences. The cortical traits were linked to reading continuously across the population, challenging categorical models of dyslexia. The findings suggest that genetic and experience-dependent mechanisms jointly shape the phonological and semantic foundations of literacy.

## Introduction

To understand neurodevelopmental variation in reading ability, it is crucial to characterize the brain’s structural substrates of reading and assess their genetic basis in typical readers (Gabrieli 2009). Reading involves mapping written letter strings onto speech sounds and meaning (Castles et al. 2018). Accordingly, different forms of dyslexia—a heritable neurocognitive difficulty in learning to read—have been linked to deficits in phonological or semantic functions (Coltheart et al. 1983; Snowling 1998). Consistent with these behavioral findings, anatomical variation has been reported in the auditory cortex and the anterior temporal lobes (ATLs), which are implicated in semantic representation (Woollams et al. 2007; Clark et al. 2014; Skeide et al. 2018). Dorsal and ventral white matter tracts connecting posterior visual and auditory regions to anterior temporal and frontal brain areas are also often atypical in dyslexic populations (Vandermosten et al. 2013; Zhao et al. 2016; Williams et al. 2018; Banfi et al. 2019). However, genetic influences on reading extend well beyond dyslexia. A meta-analysis of typical readers estimated heritability at *h*^2^ = 66% for general reading ability and *h*^2^ = 68% for “phonological decoding,” that is, the ability to find the correct pronunciation of written words (Andreola et al. 2021). Thus, microstructural variation in cortical regions and white matter pathways central to auditory and semantic functions may partly reflect a continuous, heritable variation in reading ability across the general population. The present study investigated links between reading ability and microstructural variation in the auditory cortex, ATL, and the major dorsal and ventral white matter pathways supporting phonological and semantic processing in a large sample of typical readers drawn from the Human Connectome Project Young Adult (HCP-YA) database (Van Essen et al. 2013).

Two distinct routes from written words to meaning have been proposed. These routes are thought to rely on partially distinct cortical and white matter pathways corresponding broadly to the dorsal phonological and ventral semantic streams of language processing (Hickok and Poeppel 2007). Various types of dyslexia can arise from selective disruption of these systems.

### Phonological route

#### Cortical substrates

The phonological route maps orthographic symbols to phonological representations, which in turn evoke semantic interpretations. Since it does not require prior knowledge of the written words, this pathway is particularly important at earlier stages of reading development or when reading unfamiliar words. Developmental dyslexia commonly involves impaired mapping of written symbols to sounds, which has been linked to reduced phonological awareness—the ability to recognize and manipulate speech sounds (Bradley and Bryant 1983; Snowling 1998). Consequently, the auditory cortex and its surroundings have shown anatomical differences between dyslexic and typical readers, possibly linked to the hampered phonological awareness. Reduced gray matter volume has been seen in the left superior temporal sulcus and right superior temporal gyrus (STG), overlapping with auditory belt and parabelt regions (Richlan et al. 2013). In the left Heschl’s gyrus, which hosts the auditory core, dyslexic groups have displayed a genetically associated decrease in cortical thickness and increase in myelination (Clark et al. 2014; Skeide et al. 2018; Gialluisi et al. 2021).

Even in samples of typical readers, reading skills have been shown to covary with coarse-grained cortical measures across the brain. Using a previous version of the HCP-YA dataset that included 900 subjects, Cui et al. (2018) reported a widespread correlation between gray matter volume and oral reading. As for the phonological pathway, He et al. (2013) observed visual form-to-sound association skills to be related to gray matter volume in the right STG. Association between cortical volume, surface area, and reading ability has also been found in the left Heschl’s gyrus (Kepinska et al. 2026). Gray matter volume conflates 2 genetically unrelated variables: cortical thickness and surface area (Panizzon et al. 2009). In an analysis separating cortical thickness and surface area in the HCP-YA 2017 release, cortical thickness of the left Heschl’s gyrus and left-lateralization of the surface area of the temporal poles were the only features associated with improved oral reading among all areas in the Desikan–Killiany parcellation (Roll 2024). This resonates with data from typically developing children, where the cortical thickness of Heschl’s gyrus and its vicinity predicted reading skill (Perdue et al. 2020), and the surface area of the left STG was genetically correlated with oral reading (Carrión-Castillo et al. 2023).

Gray matter density in left Heschl’s gyrus also correlated with low-frequency auditory skills, which were linked to reading (Sutherland et al. 2012). However, like gray matter volume, density is also difficult to interpret, especially in the auditory cortex, where extensive folding and myelination elevate the risk of misclassifying voxels (Sigalovsky et al. 2006). One way to address this issue is to compute the T1-weighted (T1w)/T2-weighted (T2w) ratio. It amplifies myelin contrast while suppressing signal noise, offering a microstructural complement to T1w-based cortical thickness (Glasser and Van Essen 2011). While this ratio correlates with histological increases in cortical myelin content (Glasser et al. 2014), it remains an indirect proxy that may fundamentally reflect variations in axon caliber, orientation, and cellular packing density (Arshad et al. 2017).

The brain parcellations used in previous studies were based on macroscopic anatomical folding landmarks. This limits the mechanistic interpretation of the results, given the complexity of the auditory cortex and ATL. The auditory core, occupying the central medial two-thirds of Heschl’s gyrus (Wasserthal et al. 2014), is associated with the lemniscal thalamic pathway, which carries a precise spectrotemporal representation of the auditory signal. The belt areas surrounding the auditory core are instead more related to the paralemniscal pathway, tuned to slowly changing auditory patterns (Abrams et al. 2011; Garcia et al. 2025). This distinction is particularly important for reading acquisition, where an initial stage dependent on low-frequency information has been suggested (Goswami 2011; Giraud and Poeppel 2012; Hämäläinen et al. 2012; Giraud and Ramus 2013). Specifically, rises in sound intensity might help children detect syllable boundaries, onsets, and rimes, a crucial early step in letter-to-sound mapping (Goswami 2015). The current study employed a multimodal parcellation that, in addition to cortical folding, draws on cytoarchitectonically related measures and functional imaging and connectivity results (Robinson et al. 2014; Glasser et al. 2016). This allows for a division of the auditory cortex into functionally motivated core and belt areas (Sweet et al. 2005).

#### White matter tracts

The arcuate fasciculus (AF) is widely considered a major component of the dorsal phonological pathway connecting the auditory region to motor speech areas (Catani and Thiebaut de Schotten 2008). Connecting auditory impressions to motor commands is essential to acquire speech sounds during phonological development (Novén et al. 2021). Accordingly, lower white matter fractional anisotropy (FA) in the left AF has been reported in dyslexic compared to typical readers (Klingberg et al. 2000; Vandermosten et al. 2012). The reduced FA has often been interpreted as reflecting altered axonal organization in auditory–motor connections. Further, decreased left-lateralization of AF FA has been found in dyslexia (Vandermosten et al. 2013; Banfi et al. 2019) and as a negative correlate of phonological decoding in typically developing children (Perdue et al. 2025). FA and fiber density measures in the left AF and adjacent cortex have also predicted phonological awareness and decoding ability in typically developing children (Niogi and McCandliss 2006; Yeatman et al. 2011; Cheema and Cummine 2018; Perdue et al. 2025) and adults (Klingberg et al. 2000). Moreover, the left AF has been seen to develop over time, particularly in poor decoders (Perdue et al. 2025), and its structural changes are accompanied by improved decoding (Roy et al. 2025). Although FA has not consistently shown a relation to reading in the AF (Christodoulou et al. 2017; Cheema and Cummine 2018), more sensitive diffusion measures indicate that there is a relation between axonal structure and reading skill, particularly in the temporoparietal portion of the dorsal fiber bundles (Meisler and Gabrieli 2022). Specifically, lower orientation dispersion and neurite density indices have indicated more pruned and streamlined tracts in skilled young readers (Koirala et al. 2021).

### Semantic route

#### Cortical substrates

In the semantic route, the orthographic input is instead mapped directly onto the semantic system, which then activates the linked phonological structure (Plaut et al. 1996). This pathway is particularly important for fluent reading of familiar and irregularly spelled words. Damage to the semantic pathway is present in “surface dyslexia,” which is characterized by effortful reading of words with nonregular spelling. Although surface dyslexia can also be developmental (Coltheart et al. 1983), its neurostructural correlates have been studied mostly in its acquired form. Since it is often present in semantic dementia with ATL atrophy, the primary mechanism is assumed to be disruption of the mapping from orthography to the semantic system of the ATL (Woollams et al. 2007; Wilson et al. 2009; Ueno et al. 2018). The ATL is known to function as a semantic hub, organizing and categorizing sensory features represented all over the brain into concepts (Patterson et al. 2007). In line with the semantic system’s role in reading, cortical volume along the left ventral visual stream predicted word and pseudoword reading speed and accuracy in schoolchildren (Beyer et al. 2022). More specifically, the cortical volume of the middle temporal gyrus portion of the left ATL has been linked to form-to-sound-association skills (He et al. 2013). In a macrostructural cortex parcellation, the temporal pole surface area lateralization predicted oral reading (Roll 2024). Nevertheless, the ATL can be divided into functionally distinct dorsal and ventral poles, and middle and inferior lateral areas that are heritable and present already in newborns (Rice et al. 2015; Glasser et al. 2016; Zhu et al. 2025). The dorsal temporal poles have been argued to be bilaterally involved in language processing, whereas the ventral poles are more visually related (Zhu et al. 2025). While the left anterior middle temporal gyrus is specialized in language-related semantic knowledge retrieval, the right temporal pole seems to be engaged in recognition and identification of visual objects and faces (Snowden et al. 2004; Snowden et al. 2012; Hurley et al. 2018; Landi et al. 2021). It is currently unknown how the subregions of the ATL relate to reading.

#### White matter tracts

The semantic system of the ATL interfaces with the auditory cortex and surrounding temporal networks through the middle longitudinal fasciculus (Maldonado et al. 2013) and the posterior indirect segment of the AF (Catani and Thiebaut de Schotten 2008; Basile et al. 2024). The ATL also connects to language-related frontal areas via the uncinate fasciculus (UF) and to higher-order visual areas through the inferior longitudinal fasciculus (ILF). Both pathways have been implicated in word comprehension (Del Tufo et al. 2019; Nguyen et al. 2025). Connecting visual, temporal, and frontal areas, the inferior fronto-occipital fasciculus (IFOF) plays a vital role in the semantic pathway of reading and in semantic selection at large (Giampiccolo et al. 2025). Its impairment by stimulation leads to semantic paraphasias (Duffau et al. 2005). A reduced left-lateralization of the IFOF and thinner cortex mainly along the left ventral visual stream have also been seen in dyslexia (Zhao et al. 2016; Williams et al. 2018). FA in IFOF and ILF has also shown a positive structural and maturational relation to reading performance—in particular, orthographic processing—in typically developing young schoolchildren (Yeatman et al. 2011; Cheema and Cummine 2018; Vanderauwera et al. 2018). However, complex negative and positive relations with FA have been found for children’s language comprehension in the ILF (Del Tufo et al. 2019), and for teenagers and young adults’ reading skill in the IFOF (Cheema and Cummine 2018). In line with Koirala et al. (2021), these patterns have been attributed to neural pruning.

Although diffusion measures of white matter microstructure are themselves heritable (Gustavson et al. 2019), it remains unresolved to what extent their associations with reading ability are attributable to shared genetic influences.

### The current study

Previous research has identified structural differences between individuals with and without dyslexia in the brain’s phonological and semantic pathways. The contrasts involved the core auditory regions in the superior temporal plane and semantic areas in the ATL, as well as dorsal and ventral nerve fiber tracts that support the pathways. Likewise, continuous relations between reading ability and surface area, cortical thickness, and tractography measures have been reported along these pathways. It is therefore unclear whether these cortical and white matter characteristics are specific to clinical groups or reflect continuous variation across the general population, some of which may be genetically influenced. Prior investigations of typical readers have either involved coarse-grained structural parcellations of the cortex or used measures that do not separate surface area from cortical thickness, and have not included metrics that could give information about cortical myelination. This limits mechanistic understanding of how distinct cortical subregions contribute to reading acquisition.

Here, we mapped microstructural variation within functionally distinct subregions of the auditory cortex and ATL by combining high-resolution magnetic resonance imaging (MRI), fine-grained multimodal cortical parcellation (Robinson et al. 2014; Glasser et al. 2016), and a genetically informed twin design in 1,068 young adults from the Human Connectome Project (Van Essen et al. 2013). We quantified cortical surface area, thickness, and the T1w/T2w image ratio in these subregions and used the twin design to estimate the relative contributions of heritable and experience-dependent influences. To move beyond coarse anatomical parcellations, the auditory cortex and ATL were subdivided into functionally informed regions (Glasser et al. 2016), enabling us to test whether distinct auditory and semantic subcomponents show different relationships with reading ability. We addressed 2 main questions. First, we asked whether oral reading ability is associated with microstructural variation in functionally defined auditory and anterior temporal cortical subregions and, if so, whether these associations reflect genetic or environmental influences. Second, we inquired whether analogous associations exist for the major dorsal and ventral white matter pathways (AF and IFOF) and, if so, whether they likewise reflect shared genetic influences. FA, as well as radial diffusivity (RD) and axial diffusivity (AD), were quantified along the AF and IFOF. These analyses enabled us to assess whether individual differences in reading ability are associated with heritable or experience-dependent variation in cortical regions, their connecting white matter pathways, or both.

## Materials and methods

### Participants and data source

We analyzed 3T MRI and behavioral data from the HCP-YA dataset (*N* = 1,096; 596 female; age: 22 to 37 yr/o, *M* = 28.8 ± 3.7) (Van Essen et al. 2013). Twenty-five participants with incorrect right–left phase-encoding direction were excluded in accordance with the HCP-YA 2025 data release recommendations. Participants flagged during the HCP radiologist quality control assessment were subsequently reviewed manually for atypical variation in the regions of interest (ROIs), resulting in the exclusion of 3 additional participants. The final sample comprised 1,068 participants. Cortical metrics were derived from the HCP’s minimal preprocessing pipeline (1,200 subjects 2017 release) (Glasser et al. 2013; Robinson et al. 2014). All cortical measures were analyzed in the HCP fs_LR 32k surface space, based on the Conte69 atlas (Van Essen et al. 2012). A genetically confirmed twin subsample (*N* = 396; monozygotic = 246, dizygotic = 150) was used for the cortical genetic analyses. The diffusion analyses used data from the HCP-YA 2025 release. Of the 1,037 eligible participants matching the cortical sample, one was excluded because a missing standard-to-anterior commissure–posterior commissure (ACPC) warp file precluded transformation of standard space data into native space. The final diffusion sample comprised 1,036 participants, including a twin subsample of 380 participants (242 monozygotic, 138 dizygotic).

All data acquisition procedures were performed in compliance with relevant laws and institutional guidelines and approved by Washington University in St. Louis Institutional Review Board (ID: 201204036). Participants’ privacy rights were respected, and their informed consent was obtained (Elam et al. 2021). Facial features were removed from MRI images using an automated defacing algorithm (Milchenko and Marcus 2013). HCP granted access to restricted data for this study. The data analysis procedures were approved by the Swedish Ethical Review Authority (ID: 2025-03150-01).

### MRI acquisition and processing

High-resolution T1w images were acquired by the HCP on a modified Siemens Skyra using a 3D MPRAGE sequence (iPAT2, TR = 2,400 ms, TE = 2.14 ms, flip angle = 8, 0.7 mm isotropic, acquisition time = 460 s). T2w images were acquired with a 3D T2-SPACE sequence (TR = 3,200 ms, TE = 565 ms, flip angle mode = T2 var, 0.7 mm isotropic, acquisition time = 504 s). Structural images were processed using the HCP minimal preprocessing pipeline (Glasser et al. 2013; WU-Minn 2017), including gradient nonlinearity correction, bias field correction, brain extraction, ACPC alignment, and nonlinear registration to Montreal Neurological Institute (MNI) space. Cortical surface reconstruction and morphometric measures were generated as part of the HCP Young Adult S1200 minimal preprocessing pipeline using HCP Pipelines v3.21 and FreeSurfer 5.3.0-HCP. Cortical thickness and surface area were derived from T1w images. The T1w/T2w ratio was used as a proxy for myelin content (Glasser and Van Essen 2011). Surface data were extracted and visualized using Connectome Workbench 2.0.1 (Marcus et al. 2011).

Diffusion data were acquired by the HCP in 6 sessions using a spin-echo echo-planar imaging (EPI) sequence (TR = 5,520 ms, TE = 89.5 ms, flip angle = 78, 1.25 mm isotropic, multiband factor = 3, acquisition time = 590 s). Data were collected across 3 diffusion weightings (b = 1,000, 2,000, and 3,000 s/mm^2^; 90 directions per shell) with reversed phase-encoding directions. Diffusion data were then preprocessed using the HCP minimal preprocessing pipeline (Glasser et al. 2013), including susceptibility distortion correction (topup) (Andersson et al. 2003), eddy current and motion correction (Andersson and Sotiropoulos 2016), gradient nonlinearity correction, and bias field correction. Probabilistic masks of the AF and IFOF were obtained from the 1-mm FSL XTRACT atlas defined in MNI space. Atlas maps were first transformed to each subject’s native diffusion space using a 2-step procedure: nonlinear MNI-to-T1 transformation via the HCP standard-to-ACPC warp field, followed by linear registration from T1 to diffusion space. Trilinear interpolation was used during resampling. The transformed probability maps were thresholded at 30% and binarized in diffusion space. Diffusion metrics were extracted within these subject-aligned tract masks: FA, RD, and AD.

### Regions of interest

**Figure 1:**
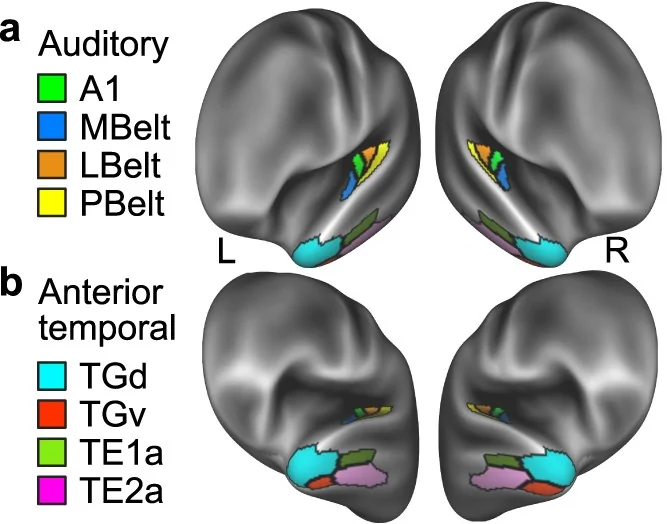
Bilateral regions of interest shown on the inflated average brain. (a) The auditory cortex includes the auditory core (A1), medial belt (MBelt), lateral belt (LBelt), and parabelt (PBelt). (b) The anterior temporal lobe comprises the dorsal (TGd) and ventral (TGv) temporal pole, and the anterior middle (TE1a) and inferior (TE2a) temporal gyri.

ROIs were defined using the multimodal cortical parcellation of Glasser et al. (2016), which integrates architecture, function, connectivity, and topography. We examined two bilateral cortical systems: (i) the auditory cortex, comprising the auditory core (A1), the medial (MBelt) and lateral belt (LBelt), and the parabelt (PBelt); and (ii) the ATL, including the dorsal (TGd) and ventral (TGv) temporal pole, and the anterior middle (TE1a) and inferior (TE2a) temporal gyri (Fig. 1).

### Behavioral tests

Reading ability was assessed using the NIH Toolbox Oral Reading Recognition Test, which evaluates pronunciation of letters and words through an adaptive procedure (*N* = 1,068; *M* = 116.9 ± 10.6, range = 84.2 to 150.7) (Gershon et al. 2013). All cortical and tract measures were assessed as predictors of reading ability (Tables S1–S4). To dissociate semantic from visuospatial functions in anterior temporal regions, we also tested bilateral surface area and cortical thickness of the final multimodal model ATL predictors (see below) against performance on the NIH Toolbox Picture Vocabulary Test (PicVocab; *N* = 1,068; *M* = 117.0 ± 9.7, range = 90.7 to 153.1) and Variable Short Penn Line Orientation Test (VSPLOT; *N* = 1,061; *M* = 14.9 ± 4.5, range = 1 to 26) in separate linear models (Table S10). PicVocab is an adaptive measure of semantic ability in which participants select the picture that best matches a spoken word, whereas VSPLOT assesses visuospatial processing by requiring participants to rotate a movable line until it appears parallel to a fixed line.

### Statistical analyses

Statistical analyses were performed in R version 4.4.3 (R Core Team 2025). All models included intracranial volume (ICV), age (in years), gender, and handedness as covariates, with age, ICV, and handedness scaled (*z*-scored and centered) for model stability. A 2-stage ROI-based approach was used to minimize collinearity while controlling for multiple comparisons. In the first stage, separate bilateral linear models were fitted for each cortical metric (thickness, surface area, and T1w/T2w ratio) predicting reading ability (Tables S1–S3). *P*-values from 2-tailed *t* tests (df = 1,047) of ROI predictors were corrected for multiple comparisons within each model using the Benjamini–Hochberg false discovery rate (FDR) procedure. Multicollinearity was assessed using variance inflation factors (VIFs), with a threshold of VIF < 3.0.

Predictors surviving FDR correction and VIF filtering (Table 1) were entered into a second-stage multimodal model (residual df = 1,057) to determine whether their associations with reading performance remained independent when considered simultaneously and to estimate the variance in oral reading performance jointly explained by the identified predictors. To increase comparability across predictor metrics, the predictor and outcome variables were scaled. The model had the formula:


\begin{align*} Readin{g}_i&={\beta}_0+{\beta}_1{\left(L\ A{1}_{thickness}\right)}_i+{\beta}_2{\left(L\ A{1}_{area}\right)}_i\nonumber\\&\quad+{\beta}_3{\left(L\ MBel{t}_{T1w/T2w}\right)}_i+{\beta}_4{\left( TE1{a}_{area}\right)}_i+{\beta}_5{\left(L\ TE2{a}_{thickness}\right)}_i\nonumber\\&\quad+{\beta}_6{\left(R\ TG{d}_{area}\right)}_i+{\gamma}^{\top }{C}_i+{\varepsilon}_i \end{align*}

where ${\gamma}^{\top }{C}_i$ denotes the regression coefficients of the covariates (ICV, handedness, age, and gender) for participant *i*. Holm correction was then applied to the predictor-specific *P*-values to control the family-wise error rate (FWE; 2-tailed; Table S6), and nonsignificant variables were excluded (Table 2). No evidence of multicollinearity (all VIF < 1.6) or heteroscedasticity (Breusch–Pagan *BP*(9) = 14.65, *P* = 0.101) was observed in the final model. For visualization purposes, left MBelt T1w/T2w values were winsorized at the first and 99th percentiles to reduce the visual influence of extreme points. All reported statistics are based on the non-winsorized data. Standardized β and model-level effect sizes (*R^2^*) are reported in the corresponding results table.

**Table 1:** Predictors surviving FDR correction in the screening models. Two predictors were excluded from the multimodal cortical model due to multicollinearity (VIF > 3). Full screening results are provided in Tables S1–S5.

**Outcome**	**Model**	**Predictor**	***B***	***P*** _***FDR***_	**Retained**
Oral Reading	Area	L A1	0.04	0.002	Yes
		L PBelt	−0.02	0.011	No (VIF > 3)
		R PBelt	0.03	0.017	No (VIF > 3)
		L TE1a	0.01	0.041	Yes
		R TGd	0.01	0.004	Yes
	Thickness	L A1	5.95	0.027	Yes
		L TE2a	−8.06	< 0.001	Yes
	T1w/T2w	L MBelt	−19.19	0.003	Yes
	FA	R AF	−65.31	0.034	Yes
	RD	L IFOF	84.24	0.013	Yes
TE2a thickness	FA	L AF	1.49	0.004	Yes

*B*, unstandardized regression coefficient.

**Table 2:** Linear models with cortical and white matter structural metrics and covariates predicting “oral reading.” Heritable (*h*^2^) and unique environmental influences (*e*^2^) on the structural traits are shown (AE model), along with the genetic correlation between each structural trait and reading (*r_g_*). All variables are scaled and mean-centered by dividing by 1 SD. Intercepts and covariates are presented in Tables S7 and S8.

**Structure**	**Metric**	**β**	***SE***	***P* (β)**	***h*** ^***2***^ **[95% CI]**	***e*** ^***2***^ **[95% CI]**	***r*** _***g***_ **[95% CI]**	***P* (*r*** _***g***_ **)**
*Cortical model*								
L A1	Thickness	0.11	0.03	<0.001^*^	0.30 [0.14–0.44]	0.70 [0.56–0.86]	0.02 [−0.23–0.26]	0.867
L MBelt	T1w/T2w	−0.11	0.03	<0.001^*^	0.29 [0.13–0.43]	0.71 [0.57–0.87]	−0.38 [−0.66–−0.14]	0.006^*^
L TE1a	Area	0.18	0.03	<0.001^*^	0.52 [0.39–0.62]	0.48 [0.38–0.61]	0.38 [0.29–0.56]	<0.001^*^
L TE2a	Thickness	−0.11	0.03	<0.001^*^	0.42 [0.27–0.54]	0.58 [0.46–0.73]	−0.17 [−0.38–0.03]	0.199
R TGd	Area	0.14	0.03	<0.001^*^	0.66 [0.56–0.75]	0.34 [0.25–0.44]	0.32 [0.15–0.47]	<0.001^*^
*White matter model*								
L IFOF	RD	0.11	0.03	0.001^*^	0.50 [0.36–0.61]	0.50 [0.39–0.64]	0.21 [0.02–0.41]	0.066
R AF	FA	−0.07	0.03	0.017^*^	0.43 [0.28–0.55]	0.57 [0.45–0.72]	−0.11 [−0.32–0.10]	0.305

^*^ *P* < 0.05. FWE-corrected *P*-values are displayed. Cortical model: Multiple *R^2^* = 0.147, adjusted *R^2^* = 0.139, *F*(9, 1058) = 20.36, *P* < 0.001, Residual SE = 0.93. White matter model: Multiple *R^2^* = 0.091, adjusted *R^2^* = 0.085, *F*(7, 1,028) = 14.8, *P* < 0.001, Residual SE = 0.96.

Two-stage procedures were also applied to test whether diffusion metrics predicted reading ability and TE2a thickness. Bilateral AF and IFOF were selected as the principal dorsal and ventral language-related tracts, respectively. To minimize multicollinearity arising from the mathematical interdependence of diffusion tensor metrics (FA, RD, and AD), separate linear models were first fitted for each metric, using the same covariates as in the cortical analyses. Predictor *P-*values were FDR-corrected within the metric-specific models (Table S4). In a second stage, predictors of reading ability surviving FDR correction were entered into a joint model including the covariates and TE2a thickness. Predictor *P-*values in the joint model were FWE-corrected (Tables 2 and S7). Because only one predictor survived the first stage in the models predicting TE2a thickness, a second-stage analysis was only performed for that outcome to report a standardized β. To replicate a previously found relation between left-lateralization of AF FA and reading skill, a lateralization index (LI) was also calculated (Desmond et al. 1995):


$$ LI=\frac{Left\ FA- Right\ FA}{Left\ FA+ Right\ FA} $$

### Quantile regression

To examine whether associations were consistent across the range of reading performance, separate quantile regression models (Koenker and Hallock 2001) were fitted including the final cortical predictors at τ = 0.25, 0.50, and 0.75 (Table S9). All predictors remained significant after FWE correction, except for left A1 thickness and right TGd surface area, which showed effects only at τ = 0.25 and 0.50.

### Genetic analyses

Heritability was estimated for all predictors of the final linear models using standard bivariate Cholesky decomposition ACE (additive genetic, common environment, unique environment) models in OpenMx 2.21.13 (Neale et al. 2016) and umx 4.30.0 (Bates et al. 2019) (df = 773 for cortical models; 741 for tract models; Table S11). All variables were scaled prior to modeling. The genetic correlation (*r_g_*) between reading and cortical/tract predictors was calculated within the twin subsample, with significance assessed by likelihood-ratio tests comparing full models to nested models in which *r_g_* was fixed to zero. Twin modeling leverages the greater genetic similarity of monozygotic (100% shared genes) than dizygotic twins (~50% shared genes) raised in comparable environments to partition variance into A, C, and E components. The genetic correlation quantifies the proportion of covariance between 2 traits *X* and *Y* attributable to shared genetic influences:


$$ {r}_g=\frac{CO{V}_A\left(X,Y\right)}{\sqrt{VA{R}_A(X)\; VA{R}_A(Y)}} $$

where *COV_A_* is the genetic covariance, and *VAR_A_* the additive genetic variance of each trait (Neale and Cardon 1992). For cortical models, constraining the shared environmental component (C) to zero did not significantly worsen model fit. The more parsimonious AE (additive genetic, unique environment) models were therefore retained (Neale and Cardon 1992) (df = 776). For tract models, the C component slightly improved the fit for right AF FA (R AF FA; *AIC_ACE_* = 2,001 and *AIC_AE_* = 2,003, *Δχ*^2^(3) = 7.98, *P* = 0.046). However, the shared environmental variance estimate for this trait was small and imprecise (*c*^2^ = 0.03, 95% CI −0.46 to 0.44), indicating limited power to distinguish the C component also in the diffusion subsample. Given the instability of the C estimate, the minimal Akaike Information Criterion (AIC) difference, and for consistency across models, the AE specification was retained for interpretation. Confidence intervals for standardized variance components and *r_g_* were obtained using profile-likelihood methods in OpenMx (CSOLNP optimizer).

## Results

**Figure 2:**
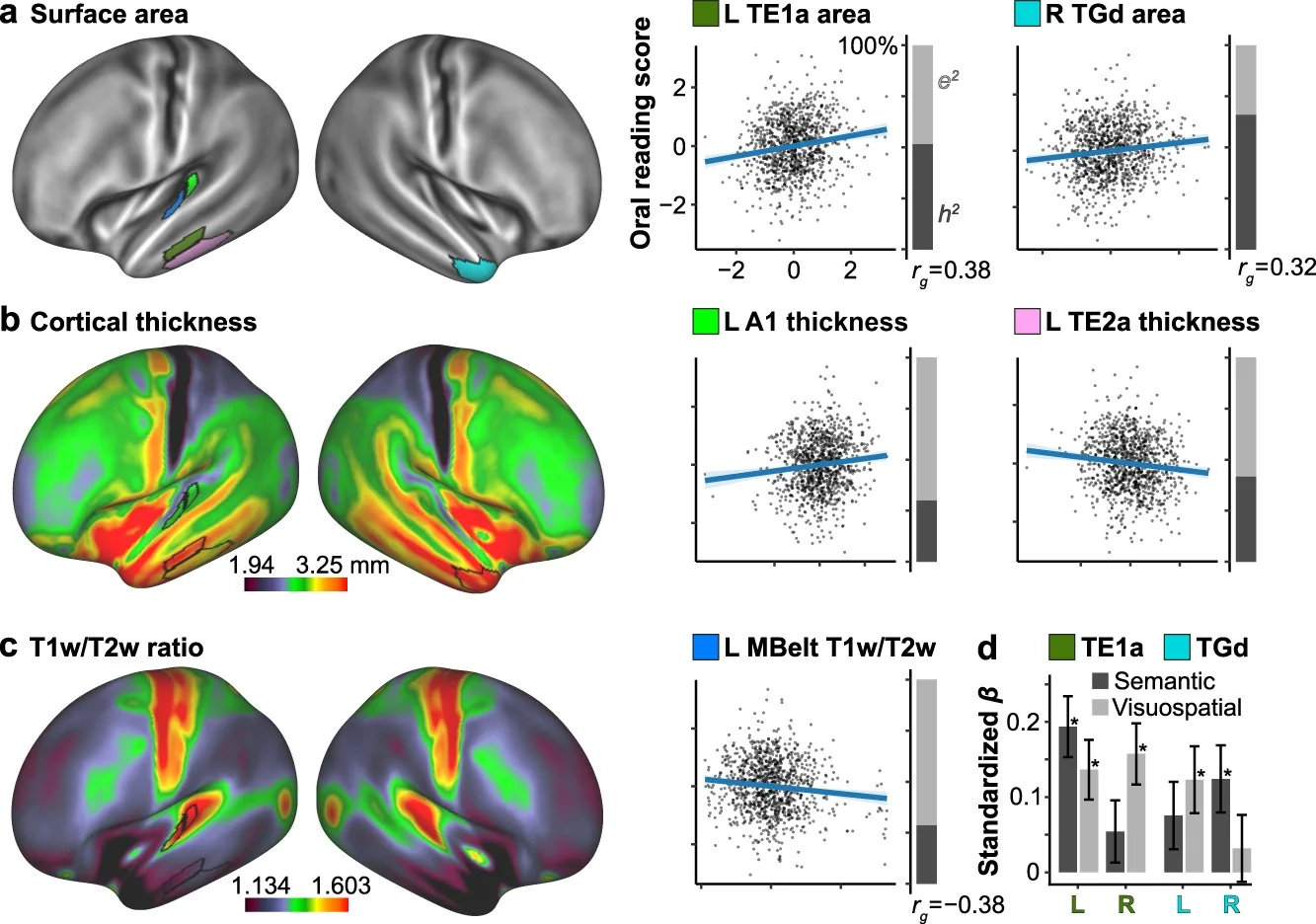
Cortical traits underlying reading ability. Group average maps of (a) surface area, (b) cortical thickness, and (C) T1w/T2w ratio projected onto the inflated brain surface. Regression lines show associations with oral reading in the final model, with individual participant scores plotted as scatter points (*N* = 1,068). Shaded areas represent 95% confidence intervals for the regression fits. Vertical bars indicate variance explained by heritability (*h^2^*) and nongenetic influences (*e^2^*). Significant genetic correlations with reading scores (*r_g_*) are labeled. (D) Anterior temporal lobe: The surface area genetically linked to reading ability was associated with semantic rather than visuospatial processing. Error bars indicate ±1 standard error of the standardized regression coefficients (β). All effects shown survive FWE correction (*P* < 0.05).

**Figure 3:**
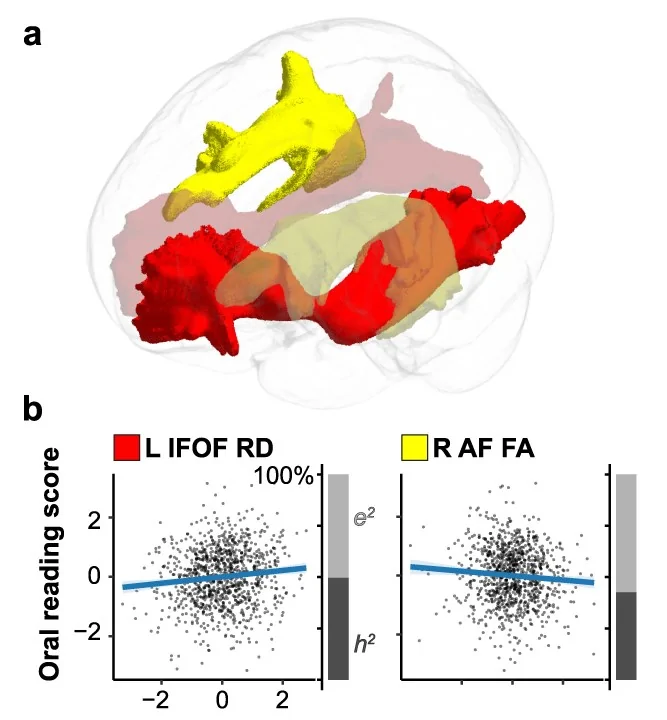
White matter pathways related to reading ability. (a) RD in the left IFOF (L IFOF; highlighted ventral pathway) and FA in the right AF (R AF; highlighted dorsal pathway) predicted reading ability, with no evidence that these associations were genetically mediated. (b) Relationship between diffusion measures and oral reading performance in the full model. Each point represents an individual participant (*N* = 1,036); the solid line denotes the fitted regression, and shaded areas indicate 95% confidence intervals. The vertical bars show the estimated genetic (*h*^2^) and nongenetic (*e*^2^) contributions to variance in L IFOF RD and R AF FA.

Oral reading ability was predicted by both heritable and experience-dependent cortical traits. Genetically mediated microstructure in the left auditory medial belt and bilateral anterior temporal semantic cortex was associated with reading skill. Experience-dependent variation within an integration network linking left auditory and anterior temporal regions via the AF and IFOF also contributed to reading performance (Figs 2 and 3; Table 2). The predictors of the screening models that survived FDR correction are shown in Table 1. Left A1 surface area was not significant in the multimodal model (*P* = 0.242) and was excluded from the final model (Table 2).

### Phonological route

In the auditory cortex, left medial belt (L MBelt) T1w/T2w ratio was negatively associated with reading skill (β = −0.11 ± 0.03, *P* < 0.001) and showed a significant genetic correlation (*r_g_* = −0.38, *P* = 0.006; Fig. 2C). The left auditory core thickness was positively associated with reading (β = 0.11 ± 0.03, *P* < 0.001; Fig. 2B). This association did not show evidence of a genetic component. Quantile regression indicated that the effect of auditory core thickness was most pronounced in lower-to-medium-performing readers (τ = 0.25, 0.50; Table S9).

### Semantic route

In the ATL, surface area in the left anterior middle temporal gyrus (L TE1a; β = 0.18 ± 0.03, *P* < 0.001; *r_g_* = 0.38, *P* < 0.001) and right dorsal temporal pole (TGd; β = 0.14 ± 0.03, *P* < 0.001; *r_g_* = 0.32, *P* < 0.001) showed a positive heritable association with oral reading (Fig. 2A). To further examine the functional significance of these regions, their association with semantic and visuospatial processing skills was tested using the PicVocab and VSPLOT tasks. Consistent with a semantic function, both areas selectively predicted performance on the semantic task in FWE-corrected post hoc tests (left TE1a: β = 0.19 ± 0.04, *P* < 0.001; right TGd: β = 0.13 ± 0.04, *P* = 0.011). In contrast, the surface area of their contralateral homologs was related to visuospatial ability (right TE1a: β = 0.16 ± 0.04, *P* < 0.001; left TGd: β = 0.12 ± 0.04, *P* = 0.010), indicating differential hemispheric specialization for semantic and nonverbal visuospatial processing (Fig. 2D; Table S10).

Cortical thickness in the left anterior inferior temporal gyrus (TE2a) was negatively related to reading scores (β = −0.11 ± 0.03, *P* < 0.001; Fig. 2B) but not to semantic processing (Table S10). This region forms part of the temporal network linking posterior visual and auditory cortical regions with frontal language areas via the anterior segments of the AF and IFOF (Baker et al. 2018; Gonzalez Alam et al. 2024). Its reduced cortical thickness may therefore index maturational optimization of long-range cortico-cortical projections, rather than local gray matter loss per se. To test this hypothesis, we modeled the fractional FA, RD, and AD of bilateral AF and IFOF. Only the left AF FA predicted TE2a thickness (β = 0.13 ± 0.04, *P* < 0.001), suggesting a structural relationship (Table S5).

### White matter tracts

Among the tested tract metrics, left IFOF RD (β = 0.11 ± 0.03, *P* = 0.001) and right AF FA (β = −0.07 ± 0.03, *P* = 0.017) were related to reading ability, independently of TE2a thickness (Fig. 3; Tables 1 and 2, Table  S4). Left IFOF RD showed genetic correlation with reading before (*P* = 0.033), but not after FWE correction (*P* = 0.066). Right AF FA did not correlate genetically with reading skill (Tables 2 and S11). We further examined the AF lateralization effect by computing the AF FA LI and its relationship with reading in a covariate-adjusted model (β = 0.07 ± 0.03, *P* = 0.021). Bilateral models showed that left (β = 0.10 ± 0.03, *P* < 0.048) but not right (*P* = 0.989) IFOF RD was related to semantic ability rather than visual (*P* = 0.812) (Table S10). To better characterize the positive RD association with reading skill, AD in the left IFOF was further examined separately in a covariate-adjusted model and was likewise positively related to reading skill (β = 0.08 ± 0.03, *P* = 0.009).

## Discussion

We investigated whether neuroanatomical variation in the phonological and semantic pathways previously associated with dyslexia extends to a continuous relationship with reading skill across the broader population. The microstructure of structurally and functionally defined subregions within core auditory and semantic cortical areas in the superior temporal plane and the ATL was assessed in typical readers from the HCP-YA dataset. We further examined the diffusion characteristics of the principal dorsal and ventral white matter tracts. These measures were related to oral reading performance, and genetic correlations between the neuroanatomical and behavioral measures were estimated. The findings reveal a refined dual-route cortical system supporting reading. They suggest that genetic architecture shapes the capacity of foundational regions, while experience sculpts areas and tracts that refine and integrate the subskills of reading. The results extend prior heritability analyses by identifying the microstructural features of specific auditory and anterior temporal subregions and complementary pathways underlying these effects. They also support the view that at least part of the neuroanatomical variation reported in dyslexia reflects the lower end of a continuum of neuroanatomical variation associated with reading ability.

### Phonological cortical route

The auditory cortex is central to the phonological route. Here, distinct cortical processing streams appear to assist reading through complementary mechanisms. The medial belt area showed a negative heritable association between T1w/T2w ratio and reading. In this area, coarse-grained paralemniscal analysis probably underlies syllable boundary and structure detection, providing a foundation for grapheme–phoneme mapping during decoding acquisition. This pathway is particularly tuned to slowly varying amplitude envelope cues (Abrams et al. 2011), such as syllable intensity rise times, linked to phonological awareness and reading development (Goswami 2011, 2015). The heritable association between the medial belt T1w/T2w ratio and poorer reading performance suggests that this measure reflects the efficiency with which paralemniscal cues are exploited. To the extent that T1w/T2w ratio indexes cortical myelin content, this pattern supports the view that increased cortical myelin may in part reflect deficient neuronal migration affecting phonological awareness and decoding ability (Giraud and Ramus 2013). In this regard, it extends reports of auditory hypermyelination in dyslexia (Skeide et al. 2018) to a relation between auditory cortex microstructure and reading skill across the broader population.

In contrast, fine-grained spectrotemporal detail necessary for precise phoneme identification depends more on the lemniscal pathway of the auditory core, which might also compensate when paralemniscal sampling is suboptimal. The positive association between auditory core thickness and reading skill was experience-dependent. This pattern aligns with prior observations that reduced auditory cortical thickness characterizes dyslexia (Clark et al. 2014). It also refines previously found coarse-grained associations between auditory cortical thickness and reading skill in typical readers (Perdue et al. 2020; Roll 2024). It likely reflects richer local circuitry supporting sub-phonemic integration acquired through experience (Harasty et al. 2003; Kanai and Rees 2011; Schremm et al. 2018; Roll 2024).

### Semantic cortical route

The semantic pathway presents a parallel nature–nurture contrast. The surface area of semantic hub subregions in the ATL correlated genetically with reading. This provides the first evidence that the anatomy of the semantic cortex in the ATLs contributes heritably to reading performance in the general population. Here, a larger surface area in the right dorsal temporal pole and left anterior middle temporal gyrus may confer a heritable advantage in semantic learning by providing expanded cortical column numbers and neuropil space (Rakic 1988; Buxhoeveden et al. 2001; Chapeton et al. 2022), thereby enhancing capacity for categorical, multimodal semantic-feature binding (Patterson et al. 2007). This heightened capacity may support more efficient semantic categorization, facilitating word learning and the development of spelling and word recognition. Moreover, the genetically influenced ATL regions showed a cross-hemispheric specialization not previously demonstrated for reading: The surface area of the reading-related subregions predicted semantic ability, whereas their contralateral homologs showed specialization for visuospatial processing.

As for functional subregional specialization, the left anterior middle temporal gyrus has consistently been implicated in lexical-semantic retrieval and multimodal semantic processing (Rice et al. 2015). A key to the right temporal pole’s importance in reading development may be its role in the recognition and identification of visual objects and faces (Snowden et al. 2004; Snowden et al. 2012; Hurley et al. 2018). Cells with this function have been discovered in the right dorsal temporal pole in macaque monkeys (Landi et al. 2021). The recognition of words and faces is similar in that both rely on the capacity to hierarchically group a set of visual features into a single object. Therefore, face recognition and word reading have been proposed to partly share their neural substrates (Dehaene et al. 2015). The right dorsal temporal pole might be important at an early stage of the acquisition of full-form reading. The same mechanism that the right dorsal temporal pole uses to retrieve information about faces and other visual objects could retrieve knowledge about visual word forms. The information may then be connected to lexical-semantic information in the left anterior middle temporal gyrus. Experience-dependent cortical thinning in the left anterior inferior temporal gyrus may reflect maturational optimization through neural pruning. This area was also structurally related to the left AF, possibly supporting phonological-semantic integration during reading.

### White matter tracts

Consistent with the anterior temporal cortical semantic complex, the structure of the main ventral stream fiber bundle, IFOF, of the left hemisphere predicted reading skill. The RD was positively related to reading ability. This might appear counterintuitive in light of the literature on other fiber tracts, where increased RD is often taken to index white matter degradation (Lövdén et al. 2010; Kumar et al. 2013) because it quantifies diffusion perpendicular to the principal fiber orientation. However, in healthy young adults, increased RD within the IFOF has been coupled with improved memory performance (Bender et al. 2016) and reading ability (Cheema and Cummine 2018). In this context, RD may reflect bundles composed of relatively thicker axons, following developmental pruning of thinner fibers (Cheema and Cummine 2018). Crossing fibers in ex vivo samples have been observed to decrease the AD in addition to the RD increase; the principal eigenvalue becomes diluted by competing orientations (Ye et al. 2021). Integral axons with larger diameters, on the other hand, would be expected to increase diffusion both perpendicular and parallel to the principal bundle direction, as seen in the present results and in line with more sensitive measures of white matter characteristics in the AF (Koirala et al. 2021). Nevertheless, without diffusion time–sensitive modeling, these mechanisms cannot be definitively distinguished (Harkins et al. 2021).

The observed lower FA for higher reading skill in the right AF may reflect compensatory recruitment. During reading acquisition, the left AF is the main phonological pathway (Cheema and Cummine 2018), and extra development of the right AF could be present in poorer readers. Adding to this interpretation, the adjacent superior longitudinal fasciculus has been found to be more pronounced in the right hemisphere in dyslexic children (Zhao et al. 2016). In the same vein and in line with prior findings in typical readers (Perdue et al. 2025), our post hoc tests linked oral reading skill to leftward FA asymmetry of the AF. Further support for compensatory mechanisms comes from the absence of genetic correlation between right AF FA and oral reading.

### A mechanistic model of reading acquisition

**Figure 4:**
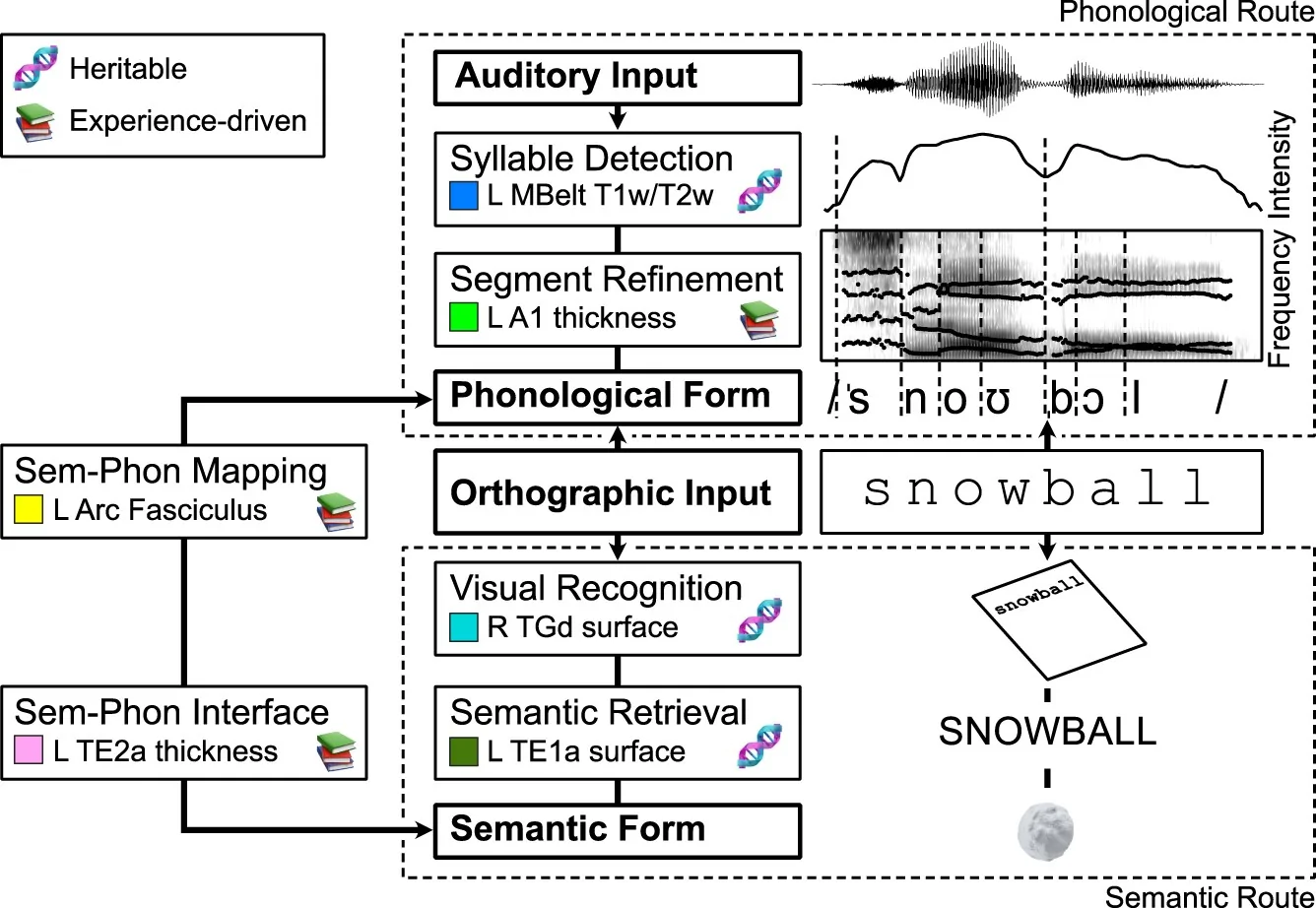
Hypothesized cortical substrates of reading acquisition. Based on the present findings, written words are proposed to be acquired through complementary phonological and semantic routes. In the phonological route, written words are associated with their corresponding auditory characteristics; in the semantic route, they are associated with visual and semantic features. Cortical traits inferred to be predominantly heritable or experience-dependent are indicated.

The current analysis moves beyond broad anatomical correlations toward a mechanistic account of the cortical system supporting reading by examining the surface area, thickness, and T1w/T2w ratio of functionally motivated subregions. A schematic hypothesis for the cortical substrates of reading acquisition is presented in Fig. 4, exemplified with the acquisition of reading the word “snowball.” The phonological route primarily supports decoding of unfamiliar written forms, whereas the semantic route increasingly builds up direct recognition of familiar words and their meanings.

In the phonological route, the auditory word is analyzed. At a first developmental stage, the paralemniscal left auditory medial belt area (L MBelt) is hypothesized to extract rather coarse-grained structure from speech based on larger contrasts in sound intensity: word- and syllable boundaries (|snow|ball|), along with onsets (sn-) and rimes (-ow) (Ferreiro and Teberosky 1979; Goswami et al. 2002). This analysis is hypothesized to depend on the cortical organization of the left medial belt (L MBelt), which may be shaped by neuronal migration during gestation. The observed negative correlation between the T1w/T2w ratio and oral reading might indicate increased myelination associated with deficient neuronal migration, as has been proposed for dyslexia (Giraud and Ramus 2013; Skeide et al. 2018), but extended to a broader population.

During reading acquisition, fine-grained spectrotemporal information is then used to refine the segmentation of individual speech sounds (/s/, /n/, /o/, /℧/…), which become associated with orthographic symbols (s-n-o-w…) (Ziegler and Goswami 2005). This process rests on the high spectrotemporal resolution of the auditory core (A1). Repeated engagement of this system may contribute to increased cortical thickness, possibly through experience-dependent modifications of dendritic arborization. The auditory system is hypothesized to project to the lexical-semantic hub via the temporal portion of the AF, which connects to the left inferior temporal gyrus (L TE2a). Increased engagement of the temporo-frontal integration network may contribute to cortical thinning in TE2a, potentially reflecting maturational optimization.

In the semantic route, seeing written words and hearing them also produces an association between word form and meaning. This is similar to the visual recognition of faces and objects and their association with semantic information about a person’s identity or an object’s characteristics. The right dorsal temporal pole (R TGd) may be particularly involved in these associations, consistent with its heritable connection to reading and its established role in the recognition of faces and visual objects. The conceptual information evoked by the written word also becomes linked to lexical-semantic representations. A greater capacity for this integration may underlie the genetic association between increased surface area of the left anterior middle temporal gyrus (TE1a) and oral reading. The semantic information can subsequently interact with phonological representations through TE2a and the AF, allowing semantic and phonological information to mutually constrain pronunciation and word recognition. The left IFOF pathway might support transmission of orthographic information to semantic representations and selection among them.

Cortical surface area was more strongly heritable than cortical thickness, and the surface areas of the ATL linked to reading were genetically conditioned. This is in line with a generally slightly higher heritability and earlier developmental expansion of cortical surface area than thickness (Panizzon et al. 2009), which shows a more protracted development through childhood, adolescence, and young adulthood (Amlien et al. 2016; Tamnes et al. 2017). T1w/T2w values were not strongly genetically influenced per se, but, to the extent they were, the variance shared with the reading ability phenotype produced a stable genetic correlation.

The results have broad implications for reading development and intervention. Because auditory and semantic traits influence decoding and comprehension along a continuum rather than in a categorical dyslexic–typical split, early support might be beneficial beyond at-risk groups. The identification of heritable semantic hubs supports semantic scaffolding (Kearns and Al Ghanem 2019) alongside phonological training as a foundation for reading development. Distinct cortical mechanisms suggest that effective interventions may combine phonological and semantic activities adapted to the diverse neurocognitive profiles encountered in classrooms.

## Supplementary Material

Supplementary_materials_bhag125

## Data Availability

Preprocessed HCP Young Adult MRI data are available from the BALSA repository (https://balsa.wustl.edu/reference/pkXDZ). The full dataset (1.57 GB) can be downloaded, or individual files can be selected as needed. Minimally preprocessed HCP Young Adult structural (1.5 TB) and diffusion (2.7 TB) MRI, as well as behavioral and demographic data, are available from the Human Connectome Project BALSA repository (https://balsa.wustl.edu/project?project=HCP_YA). Use the “Export CSV” option to download behavioral data (3.5 MB for unrestricted data, 4.3 MB including restricted variables), or select specific columns as required. All code and scripts used for the analyses are available on GitHub (https://github.com/mikaelroll/corticalreading).
