# Supplementary material for: Heritable and experience-dependent cortical traits of reading ability
Source: Cereb Cortex. 2026 Aug 22;36(8):bhag125. doi: 10.1093/cercor/bhag125 (PMC13575274; doi:10.1093/cercor/bhag125)
Supplement: Supplementary file 1 [file supplementary_materials_bhag125.pdf]

Supplementary Tables

Heritable and experience-dependent cortical traits of reading ability

Mikael Roll*

Centre for Languages and Literature, Lund University, Lund, Sweden

*Lund University, Centre for Languages and Literature, Box 201,
221 00 Lund, Sweden, mikael.roll@ling.lu.se

Screening models

Table S1 Linear model with cortical surface area predicting oral reading performance

Region	*B*	*SE*	*t*	*p*	p_{FDR}	VIF
L A1	0.041	0.011	3.78	< 0.001	0.002*	2.22
L MBelt	−0.012	0.010	-1.20	0.230	0.302	2.32
L LBelt	−0.003	0.014	-0.24	0.811	0.897	2.42
L PBelt	−0.023	0.008	-3.03	0.003	0.011*	3.67†
R A1	−0.022	0.012	-1.78	0.075	0.128	1.98
R MBelt	−0.023	0.011	-2.07	0.038	0.080	2.25
R LBelt	0.000	0.013	0.02	0.986	0.986	2.97
R PBelt	0.030	0.011	2.83	0.005	0.017*	3.53†
L TE1a	0.012	0.005	2.47	0.014	0.041*	2.25
L TE2a	0.005	0.003	1.89	0.059	0.113	2.58
L TGd	0.002	0.002	0.87	0.387	0.451	2.84
L TGv	−0.001	0.007	-0.16	0.874	0.918	1.80
R TE1a	0.009	0.006	1.56	0.118	0.165	2.47
R TE2a	0.005	0.003	1.58	0.115	0.165	2.79
R TGd	0.007	0.002	3.45	< 0.001	0.004*	2.82
R TGv	−0.010	0.004	-2.32	0.021	0.054	1.84
Intercept and covariates						
Intercept	90.46	3.659	24.7	< 0.001	NA	NA
ICV (scaled)	1.021	0.472	2.16	0.031	NA	2.47
Handedness (scaled)	0.323	0.307	1.05	0.293	NA	1.03
Age (scaled)	1.060	0.313	3.38	< 0.001	NA	1.10
Gender	−1.406	0.800	-1.76	0.079	NA	1.76

*$p < 0.05$, FDR-corrected, †VIF > 3. Scaled variables are mean-centered and divided by one standard deviation.
Multiple R^2 = 0.161, *adjusted* R^2 = 0.145, $F(20, 1047) = 9.81$, $p < 0.001$, *Residual SE* = 9.81.

Table S2 Linear model with cortical thickness predicting oral reading performance.

Region	*B*	*SE*	*t*	*p*	p_{FDR}	VIF
L A1	5.954	2.123	2.80	0.005	0.027*	1.80
L MBelt	1.771	2.239	0.79	0.429	0.600	1.77
L LBelt	−3.223	1.925	−1.67	0.094	0.283	1.75
L PBelt	−0.759	2.064	−0.37	0.713	0.788	1.74
R A1	1.708	2.031	0.84	0.401	0.600	1.73
R MBelt	−2.248	2.142	−1.05	0.294	0.600	1.67
R LBelt	1.496	1.874	0.80	0.425	0.600	1.68
R PBelt	0.405	1.988	0.20	0.839	0.881	1.72
L TE1a	−0.730	1.829	−0.40	0.690	0.788	1.50
L TE2a	−8.061	2.108	−3.82	< 0.001	< 0.001*	1.52
L TGd	2.662	2.565	1.04	0.299	0.600	1.99
L TGv	−1.243	1.429	−0.87	0.385	0.600	1.33
R TE1a	2.712	1.782	1.52	0.128	0.337	1.51
R TE2a	0.313	2.309	0.14	0.892	0.892	1.55
R TGd	−2.105	2.354	−0.89	0.371	0.600	1.93
R TGv	0.809	1.825	0.44	0.658	0.788	1.45
Intercept and covariates						
Intercept	119.0	8.375	14.2	< 0.001	NA	NA
ICV (scaled)	2.855	0.413	6.91	< 0.001	NA	1.75
Handedness (scaled)	0.569	0.318	1.79	0.074	NA	1.03
Age (scaled)	0.706	0.322	2.19	0.029	NA	1.08
Gender	−0.570	0.827	−0.69	0.491	NA	1.74

$^*p < 0.05$, FDR-corrected. Scaled variables are mean-centered and divided by one standard deviation.
Multiple $R^2 = 0.094$, *adjusted* $R^2 = 0.077$, $F(20, 1047) = 5.42$, $p < 0.001$, *Residual SE* = 10.20.

Table S3 Linear model with T1w/T2w ratio predicting oral reading performance.

Region	*B*	*SE*	*t*	*p*	p_{FDR}	VIF
L A1	−2.368	6.051	−0.39	0.696	0.730	1.39
L MBelt	−19.19	5.241	−3.66	< 0.001	0.003*	1.36
L LBelt	−18.53	8.081	−2.29	0.022	0.093	1.47
L PBelt	21.16	11.697	1.81	0.071	0.212	1.27
R A1	5.645	7.450	0.76	0.449	0.561	1.33
R MBelt	−5.115	6.837	−0.75	0.455	0.561	1.49
R LBelt	−8.031	7.370	−1.09	0.276	0.483	1.48
R PBelt	−10.90	9.699	−1.12	0.261	0.483	1.22
L TE1a	−18.84	18.687	−1.01	0.314	0.489	1.19
L TE2a	−14.57	22.589	−0.65	0.519	0.574	1.16
L TGd	−36.46	24.964	−1.46	0.144	0.337	1.18
L TGv	−7.416	10.674	−0.69	0.487	0.569	1.11
R TE1a	−15.55	16.205	−0.96	0.338	0.489	1.11
R TE2a	−5.479	20.367	−0.27	0.788	0.788	1.15
R TGd	61.26	28.323	2.16	0.031	0.108	1.16
R TGv	−18.08	15.447	−1.17	0.242	0.483	1.15
Intercept and covariates						
Intercept	242.0	78.35	3.09	0.002	NA	NA
ICV (scaled)	2.800	0.408	6.87	< 0.001	NA	1.70
Handedness (scaled)	0.550	0.318	1.73	0.084	NA	1.03
Age (scaled)	0.866	0.334	2.60	0.010	NA	1.15
Gender	−0.778	0.830	−0.94	0.349	NA	1.76

*$p < 0.05$, FDR-corrected. Scaled variables are mean-centered and divided by one standard deviation.
Multiple $R^2 = 0.095$, *adjusted* $R^2 = 0.078$, $F(20, 1047) = 5.50$, $p < 0.001$, *Residual SE* = 10.19.

Table S4 Linear models with fractional anisotropy (FA), radial diffusivity (RD), and axial diffusivity (AD) of the left inferior fronto-occipital fasciculus (L IFOF) and right arcuate fasciculus (R AF) and covariates predicting oral reading performance.

Metric	Predictor	*B*	*SE*	*t*	*p*	p_{FDR}	VIF
FA	L AF	35.70	25.41	1.41	0.160	0.214	1.70
	R AF	− 65.31	24.74	−2.64	0.008	0.034*	1.65
	L IFOF	− 61.74	27.48	−2.25	0.025	0.050	2.37
	R IFOF	9.64	24.13	0.40	0.690	0.690	2.49
	Intercept and covariates						
	Intercept	137.4	7.66	17.9	< 0.001	NA	NA
	ICV (scaled)	2.82	0.44	6.43	< 0.001	NA	1.88
	Handedness (scaled)	0.49	0.32	1.53	0.127	NA	1.02
	Age (scaled)	0.70	0.33	2.11	0.035	NA	1.08
	Gender	− 1.04	0.86	−1.21	0.226	NA	1.83
RD ($\times10^{-3}$ mm²/s)	L AF	− 45.78	29.49	−1.55	0.121	0.212	1.92
	R AF	43.06	30.57	1.41	0.159	0.212	1.92
	L IFOF	84.24	28.52	2.95	0.003	0.013*	2.88
	R IFOF	− 27.78	26.70	−1.04	0.298	0.298	2.88
	Intercept and covariates						
	Intercept	95.60	10.78	8.87	< 0.001	NA	NA
	ICV (scaled)	2.55	0.43	5.92	< 0.001	NA	1.82
	Handedness (scaled)	0.45	0.32	1.41	0.159	NA	1.02
	Age (scaled)	0.68	0.34	2.00	0.045	NA	1.15
	Gender	− 0.34	0.84	−0.41	0.682	NA	1.72
AD ($\times10^{-3}$ mm²/s)	L AF	2.41	22.91	0.11	0.916	0.916	2.89
	R AF	− 17.50	23.75	−0.74	0.461	0.916	2.66
	L IFOF	54.96	28.64	1.92	0.055	0.221	3.06
	R IFOF	− 5.10	26.34	−0.19	0.847	0.916	2.88
	Intercept and covariates						
	Intercept	99.08	11.30	8.77	< 0.001	NA	NA
	ICV (scaled)	2.67	0.43	6.20	< 0.001	NA	1.80
	Handedness (scaled)	0.52	0.32	1.61	0.107	NA	1.02
	Age (scaled)	0.69	0.33	2.09	0.037	NA	1.08
	Gender	0.00	0.87	0.01	0.996	NA	1.85

*$p < 0.05$, FDR-corrected within models. Scaled variables are mean-centered and divided by one standard deviation.
FA: *Multiple* $R^2 = 0.078$, *adjusted* $R^2 = 0.071$, $F(8, 1027) = 10.91$, $p < 0.001$, *Residual SE* = 10.20.
RD: *Multiple* $R^2 = 0.077$, *adjusted* $R^2 = 0.070$, $F(8, 1027) = 10.75$, $p < 0.001$, *Residual SE* = 10.21.
AD: *Multiple* $R^2 = 0.072$, *adjusted* $R^2 = 0.064$, $F(8, 1027) = 9.91$, $p < 0.001$, *Residual SE* = 10.24.

Table S5 Linear models with fractional anisotropy (FA), radial diffusivity (RD), and axial diffusivity (AD) of the left inferior fronto-occipital fasciculus (L IFOF) and right arcuate fasciculus (R AF), and covariates predicting left TE2a thickness.

Metric	Predictor	*B*	*SE*	*t*	*p*	p_{FDR}	VIF
FA	L AF	1.49	0.45	3.33	< 0.001	0.004*	1.70
	R AF	−0.84	0.43	−1.93	0.054	0.085	1.65
	L IFOF	0.69	0.48	1.44	0.151	0.151	2.37
	R IFOF	−0.79	0.42	−1.86	0.064	0.085	2.49
	Intercept and covariates						
	Intercept	2.80	0.13	20.8	< 0.001	NA	NA
	ICV (scaled)	0.02	0.01	2.07	0.039	NA	1.88
	Handedness (scaled)	0.00	0.01	0.53	0.599	NA	1.02
	Age (scaled)	−0.00	0.01	−0.86	0.389	NA	1.08
	Gender	0.02	0.02	1.23	0.219	NA	1.83
RD (mm²/s)	L AF	30	520	0.06	0.954	0.954	1.92
	R AF	758	539	1.41	0.160	0.616	1.92
	L IFOF	−370	503	−0.74	0.462	0.616	2.88
	R IFOF	382	471	0.81	0.418	0.616	2.88
	Intercept and covariates						
	Intercept	2.61	0.19	13.7	< 0.001	NA	NA
	ICV (scaled)	0.02	0.01	2.82	0.005	NA	1.82
	Handedness (scaled)	0.00	0.01	0.53	0.598	NA	1.02
	Age (scaled)	−0.00	0.01	−0.07	0.947	NA	1.15
	Gender	0.02	0.01	1.14	0.257	NA	1.72
AD (mm²/s)	L AF	815	402	2.03	0.043	0.165	2.89
	R AF	61	417	0.15	0.884	0.884	2.66
	L IFOF	−874	502	−1.74	0.082	0.165	3.06
	R IFOF	513	462	1.11	0.267	0.356	2.88
	Intercept and covariates						
	Intercept	2.600	0.20	13.1	< 0.001	NA	NA
	ICV (scaled)	0.017	0.01	2.29	0.022	NA	1.80
	Handedness (scaled)	0.003	0.01	0.45	0.654	NA	1.02
	Age (scaled)	−0.000	0.01	−0.05	0.958	NA	1.08
	Gender	0.014	0.02	0.91	0.363	NA	1.85

*$p < 0.05$, FDR-corrected within models. Scaled variables are mean-centered and divided by one standard deviation.
FA: *Multiple* R^2 = 0.038, *adjusted* R^2 = 0.031, $F(8, 1027) = 5.14$, $p < 0.001$, *Residual SE* = 0.179.
RD: *Multiple* R^2 = 0.029, *adjusted* R^2 = 0.021, $F(8, 1027) = 3.82$, $p < 0.001$, *Residual SE* = 0.180.
AD: *Multiple* R^2 = 0.033, *adjusted* R^2 = 0.026, $F(8, 1027) = 4.40$, $p < 0.001$, *Residual SE* = 0.180.

Multimodal models

Table S6 Multimodal linear model predicting oral reading performance.

Predictor	β	*SE*	*t*	*p*	p_{FWE}	VIF
L A1 thickness	0.107	0.029	3.67	< 0.001	< 0.001*	1.07
L A1 area	0.038	0.032	1.17	0.237	0.242	1.28
L MBelt T1w/T2w	−0.107	0.029	−3.73	< 0.001	< 0.001*	1.03
L TE1a area	0.173	0.032	5.09	< 0.001	< 0.001*	1.42
L TE2a thickness	−0.108	0.028	−3.60	< 0.001	< 0.001*	1.11
R TGd area	0.135	0.034	3.84	< 0.001	< 0.001*	1.54
Intercept and covariates						
Intercept	0.043	0.044	0.97	0.332	NA	NA
ICV	0.127	0.043	2.97	0.003	NA	2.26
Handedness	0.044	0.029	1.52	0.127	NA	1.03
Age	0.091	0.029	3.09	0.002	NA	1.08
Gender	−0.094	0.075	−1.26	0.211	NA	1.71

*$p < 0.05$, FWE-corrected. Continuous variables are scaled (mean-centered and divided by one standard deviation). *Multiple* R^2 = 0.148, *adjusted* R^2 = 0.140, $F(10, 1057) = 18.32$, $p < 0.001$, *Residual SE* = 0.93.

Table S7 Joint linear model including right arcuate fasciculus fractional anisotropy (R AF FA), left inferior fronto-occipital fasciculus radial diffusivity (L IFOF RD), and cortical thickness in left TE2a cortical thickness predicting oral reading.

Predictor	Metric	β	*SE*	*t*	*p*	p_{FWE}	VIF
R AF	FA	−0.074	0.031	−2.41	0.016	0.016*	1.07
L IFOF	RD	0.108	0.031	3.45	<0.001	0.001*	1.10
L TE2a	Thickness	−0.110	0.030	−3.64	<0.001	< 0.001*	1.02
Intercept and covariates							
Intercept		0.024	0.046	0.51	0.609		NA
ICV		0.274	0.041	6.66	<0.001		1.88
Handedness		0.049	0.030	1.62	0.107		1.02
Age		0.071	0.031	2.31	0.021		1.06
Gender		−0.054	0.078	−0.69	0.489		1.71

*$p < 0.05$, FWE-corrected. Continuous predictors and outcomes were mean-centered and divided by one standard deviation. *Multiple* R^2 = 0.091, *adjusted* R^2 = 0.085, $F(7, 1028) = 14.8$, $p < 0.001$, *Residual SE* = 0.957.

Table S8 Intercept and covariates of cortical linear model presented in Table 2.

Predictor	β	*SE*	*p*
Intercept	0.04	0.04	0.348
ICV	0.14	0.04	0.001
Handedness	0.05	0.03	0.109
Age in years	0.09	0.03	0.002
Gender	−0.09	0.07	0.227

Sensitivity analyses

Table S9 Quantile regression models with cortical predictors of reading performance and covariates.

Trait	τ	β	*SE*	*t*	*p*	p_{FWE}
L A1 thickness	0.25	0.14	0.03	4.37	< 0.001	< 0.001*
	0.50	0.10	0.04	2.34	0.019	0.019*
	0.75	0.05	0.04	1.32	0.186	0.186
L MBelt T1w/T2w	0.25	−0.15	0.03	−5.31	< 0.001	< 0.001*
	0.50	−0.13	0.05	−2.68	0.009	0.015*
	0.75	−0.09	0.04	−2.61	0.010	0.036*
L TE1a area	0.25	0.15	0.04	3.83	< 0.001	< 0.001*
	0.50	0.15	0.05	3.06	0.002	0.009*
	0.75	0.15	0.04	3.45	< 0.001	0.003*
L TE2a thickness	0.25	−0.08	0.03	−2.41	0.017	0.016*
	0.50	−0.13	0.04	−3.05	0.002	0.009*
	0.75	−0.10	0.04	−2.47	0.014	0.041*
R TGd area	0.25	0.19	0.04	4.70	< 0.001	< 0.001*
	0.50	0.16	0.05	3.31	< 0.001	0.005*
	0.75	0.09	0.04	1.99	0.047	0.095
Intercept and covariates						
Intercept	0.25	−0.55	0.04	−12.3	< 0.001	
	0.50	0.07	0.06	1.09	0.275	
	0.75	0.65	0.06	11.8	< 0.001	
ICV	0.25	0.19	0.05	4.17	< 0.001	
	0.50	0.13	0.06	2.19	0.030	
	0.75	0.06	0.06	1.05	0.295	
Handedness	0.25	0.02	0.02	1.23	0.219	
	0.50	0.06	0.04	1.57	0.117	
	0.75	0.08	0.04	2.32	0.021	
Age	0.25	0.14	0.03	4.16	< 0.001	
	0.50	0.09	0.04	2.09	0.037	
	0.75	0.02	0.04	0.45	0.650	
Gender	0.25	−0.24	0.09	−2.78	0.006	
	0.50	−0.09	0.11	−0.88	0.377	
	0.75	0.07	0.10	0.70	0.486	

*$p < 0.05$, FWE-corrected within quantiles (τ = 0.25, 0.50, and 0.75).

Table S10 Anterior temporal lobe and IFOF: Cortical metrics/RD and covariates predicting semantic (*N* = 1069) or visuospatial (*N* = 1062) performance.

Predictor	Task	Predictor	Hemisphere	*β*	*SE*	*t*	*p*	p_{FWE}
TE1a	Semantic	Area	L	0.193	0.040	4.77	< 0.001	< 0.001*
			R	0.053	0.041	1.29	0.197	0.394
	Visual		L	0.137	0.040	3.44	< 0.001	0.001*
			R	0.158	0.041	3.88	< 0.001	< 0.001*
TE2a	Semantic	Thickness	L	−0.030	0.033	−0.89	0.373	0.747
			R	−0.019	0.033	−0.57	0.567	1.000
	Visual		L	−0.065	0.033	−1.98	0.048	0.097
			R	−0.002	0.033	−0.05	0.961	1.000
TGd	Semantic	Area	L	0.071	0.045	1.58	0.115	0.230
			R	0.127	0.045	2.83	0.005	0.010*
	Visual		L	0.124	0.045	2.76	0.006	0.012*
			R	0.031	0.045	0.71	0.481	0.962
IFOF	Semantic	RD	L	0.104	0.046	2.25	0.024	0.048*
			R	0.001	0.047	0.01	0.989	0.989
	Visual		L	0.012	0.046	0.27	0.791	0.812
			R	0.039	0.047	0.83	0.406	0.812
Intercepts and covariates								
TE1a	Semantic	Intercept		0.052	0.045	1.16	0.247	
		ICV		0.171	0.040	4.28	< 0.001	
		Handedness		0.006	0.029	0.20	0.841	
		Age		0.169	0.030	5.66	< 0.001	
		Gender		−0.113	0.076	−1.50	0.135	
	Visual	Intercept		−0.014	0.044	−0.31	0.757	
		ICV		0.141	0.040	3.55	< 0.001	
		Handedness		−0.026	0.029	−0.91	0.362	
		Age		−0.015	0.029	−0.50	0.618	
		Gender		0.032	0.075	0.42	0.671	
TE2a	Semantic	Intercept		0.029	0.046	0.63	0.530	
		ICV		0.274	0.038	7.19	< 0.001	
		Handedness		0.015	0.030	0.50	0.621	
		Age		0.145	0.030	4.79	< 0.001	
		Gender		−0.061	0.077	−0.80	0.425	
	Visual	Intercept		−0.045	0.045	−1.00	0.317	
		ICV		0.268	0.038	7.06	< 0.001	
		Handedness		−0.019	0.030	−0.62	0.533	

		Age	−0.040	0.030	−1.33	0.185
		Gender	0.101	0.076	1.32	0.188
TGd	Semantic	Intercept	0.039	0.045	0.87	0.386
		ICV	0.172	0.042	4.11	< 0.001
		Handedness	0.010	0.030	0.33	0.741
		Age	0.149	0.030	5.00	< 0.001
		Gender	−0.086	0.076	−1.12	0.261
	Visual	Intercept	−0.033	0.045	−0.74	0.459
		ICV	0.185	0.042	4.41	< 0.001
		Handedness	−0.023	0.030	−0.78	0.434
		Age	−0.036	0.030	−1.22	0.223
		Gender	0.074	0.076	0.98	0.329
IFOF	Semantic	Intercept	0.017	0.046	0.38	0.706
		ICV	0.238	0.040	5.94	< 0.001
		Handedness	0.005	0.030	1.15	0.088
		Age	0.158	0.031	5.16	< 0.001
		Gender	−0.037	0.078	−0.48	0.632
	Visual	Intercept	−0.055	0.046	−1.19	0.234
		ICV	0.227	0.040	5.63	< 0.001
		Handedness	−0.014	0.030	−0.46	0.643
		Age	−0.044	0.031	−1.42	0.155
		Gender	0.114	0.078	1.46	0.144

*$p < 0.05$, after FWE-correction within each model. Continuous predictors and outcomes were mean-centered and divided by one standard deviation.
TE1a semantic: *Multiple* R^2 = 0.110, *adjusted* R^2 = 0.105, $F(6, 1061)$ = 21.92, $p < 0.001$, *Residual SE* = 0.95.
TE1a visuospatial: *Multiple* R^2 = 0.143, *adjusted* R^2 = 0.138, $F(6, 1054)$ = 29.24, $p < 0.001$, *Residual SE* = 0.93.
TE2a semantic: *Multiple* R^2 = 0.073, *adjusted* R^2 = 0.067, $F(6, 1061)$ = 13.81, $p < 0.001$, *Residual SE* = 0.96.
TE2a visuospatial: *Multiple* R^2 = 0.096, *adjusted* R^2 = 0.091, $F(6, 1054)$ = 18.62, $p < 0.001$, *Residual SE* = 0.95.
TGd semantic: *Multiple* R^2 = 0.094, *adjusted* R^2 = 0.089, $F(6, 1061)$ = 18.42, $p < 0.001$, *Residual SE* = 0.95.
TGd visuospatial: *Multiple* R^2 = 0.107, *adjusted* R^2 = 0.102, $F(6, 1054)$ = 21.01, $p < 0.001$, *Residual SE* = 0.95.
IFOF semantic: *Multiple* R^2 = 0.086, *adjusted* R^2 = 0.081, $F(6, 1029)$ = 16.14, $p < 0.001$, *Residual SE* = 0.96.
IFOF visuospatial: *Multiple* R^2 = 0.087, *adjusted* R^2 = 0.082, $F(6, 1026)$ = 16.38, $p < 0.001$, *Residual SE* = 0.96.

Genetic analyses

Table S11 Genetic Cholesky decomposition ACE models of neural traits and oral reading. The heritability of the neural trait (h^2_{ROI}) and reading (h^2_{READ}) and their genetic correlation (r_g) are shown, along with the unique environmental influences (e^2_{ROI} and e^2_{READ}).

Trait	AIC_{ACE}	AIC_{AE}	$\Delta\chi^2$	p	h^2_{ROI} [95% CI]	h^2_{READ} [95% CI]	e^2_{ROI} [95% CI]	e^2_{READ} [95% CI]	r_g [95% CI]	p	p_{FWE}
L A1 thickness	2114	2113	4.60	0.204	0.30 [0.14–0.44]	0.72 [0.63–0.78]	0.70 [0.56–0.86]	0.28 [0.22–0.37]	0.02 [−0.23–0.26]	0.859	0.859
L MBelt T1w/T2w	2107	2105	4.82	0.185	0.29 [0.13–0.43]	0.72 [0.64–0.79]	0.71 [0.57–0.87]	0.28 [0.21–0.36]	−0.38 [−0.66–−0.14]	0.002	0.006*
L TE1a area	1977	1974	3.07	0.381	0.52 [0.39–0.62]	0.72 [0.63–0.78]	0.48 [0.38–0.61]	0.28 [0.22–0.37]	0.38 [0.29–0.56]	< 0.001	< 0.001*
R TGd area	1906	1905	5.87	0.118	0.66 [0.56–0.75]	0.72 [0.63–0.79]	0.34 [0.25–0.44]	0.28 [0.21–0.37]	0.32 [0.15–0.47]	< 0.001	< 0.001*
L TE2a thickness	2087	2084	2.84	0.418	0.42 [0.27–0.54]	0.72 [0.63–0.78]	0.58 [0.46–0.73]	0.28 [0.22–0.37]	−0.17 [−0.37–0.04]	0.105	0.210
R AF FA	2001	2003	7.98	0.046	0.43 [0.28–0.55]	0.72 [0.64–0.79]	0.57 [0.45–0.72]	0.28 [0.21–0.36]	−0.11 [-0.32–0.10]	0.305	0.305
L IFOF RD	1950	1950	5.91	0.116	0.50 [0.36–0.61]	0.73 [0.64–0.79]	0.50 [0.39–0.64]	0.27 [0.21–0.36]	0.21 [0.02–0.41]	0.033	0.066

*$p < 0.05$, FWE-corrected across cortical and tract models separately. Models are controlled for intracranial volume, handedness, age, and gender. All continuous variables were scaled.
